# A Study of 5-Fluorouracil Desorption from Mesoporous Silica by RP-UHPLC

**DOI:** 10.3390/molecules24071317

**Published:** 2019-04-03

**Authors:** Monika Šuleková, Lucia Váhovská, Alexander Hudák, Lukáš Žid, Vladimír Zeleňák

**Affiliations:** 1 Department of Chemistry, Biochemistry and Biophysics, Institute of Pharmaceutical Chemistry, The University of Veterinary Medicine and Pharmacy in Košice, 041 81 Košice, Slovakia; alexander.hudak@uvlf.sk; 2 Department of Chemistry, Biochemistry and Biophysics, Institute of Biochemistry, The University of Veterinary Medicine and Pharmacy in Košice, 041 81 Košice, Slovakia; lucia.vahovska@uvlf.sk; 3 Department of Inorganic Chemistry, Faculty of Sciences, P. J. Šafárik University, Moyzesova 11, SK-041 54 Košice, Slovakia; lukas.zid@student.upjs.sk (L.Ž.); vladimir.zelenak@upjs.sk (V.Z.)

**Keywords:** mesoporous silica, 5-fluorouracil, desorption, RP-UHPLC

## Abstract

In cancer treatment, the safe delivery of the drug to the target tissue is an important task. 5-fluorouracil (5-FU), the well-known anticancer drug, was encapsulated into the pores of unmodified mesoporous silica SBA-15, as well as silica modified with 3-aminopropyl and cyclohexyl groups. The drug release studies were performed in two different media, in a simulated gastric fluid (pH = 2) and in a simulated body fluid (pH = 7) by RP-UHPLC. The simple and rapid RP-UHPLC method for quantitative determination of 5-fluorouracil released from unmodified and modified mesoporous silica SBA-15 was established on ODS Hypersil C18 column (150 × 4.6 mm, 5 µm) eluted with mobile phase consisted of methanol: phosphate buffer in volume ratio of 3:97 (*v*/*v*). Separation was achieved by isocratic elution. The flow rate was kept at 1 mL/min, the injection volume was set at 20 µL and the column oven temperature was maintained at 25 °C. The effluent was monitored at 268 nm. This paper provides information about the quantitative determination of the released 5-FU from silica. It was found out that larger amount of the drug was released in neutral pH in comparison with the acidic medium. In addition, surface functionalisation of silica SBA-15 influences the release properties of the drug.

## 1. Introduction

Over the past three decades, there has been rapid growth in the area of silica-based ordered mesoporous materials as drug delivery systems owing to the great versatility and stability of these mesoporous matrices. MCM-41, MCM-48 and SBA-15 are the most common mesoporous silica materials with the pore size ranging from 2–10 nm and 2D-hexagonal and 3D-cubic structural characteristics [1]. The high surface area, tunable pore diameter, good biocompatibility/biodegradation [2,3] and uniform mesoporous structure of the mesoporous silicas allow the adsorption of drugs and biomolecules into their mesostructures to be then locally released. There are also triggers that can activate the release of the guest molecules, such as heat, pH, light, chemicals, ultrasound or even magnetism [4]. In the family of mesoporous silica materials, SBA-15 silica was studied more than the others, due to its higher hydrothermal stability [5].

The use of silica-based ordered mesoporous materials as drug delivery systems was carried out for the first time in 2001. The first to describe the use of mesoporous silica as drug delivery support were Regí et al. [6], who enclosed ibuprofen in a MCM-41 matrix in an effort to design an implantable delivery system. Three years later, controlled release of gentamicine sulphate as model drug from SBA-15 was studied by a HPLC method [7]. In addition, the antibiotic amoxicillin has been incorporated into the porous calcined SBA-15 by contacting a solution of the drug in an appropriate solvent with the porous solid. The drug release process from the matrix into a simulated body fluid solution has been studied [8]. In other works, the release of nimodipine from SBA-15 [9], naproxen from MCM-41 [10] and SBA-15 [11], as well as indomethacin from MCM-48 mesoporous silica were studied, too [12].

A great advantage of silica-based ordered mesoporous materials (SMMs) is that these materials can be modified by various functional groups. The surface of SMMs presents a high density of silanol groups, which are also susceptible of undergoing a chemical modification with a large variety of organic groups through functionalization process [13]. Thus, it is possible to modulate the chemical properties of the mesoporous surfaces to achieve the desired properties [14,15,16,17]. For example, modification by various functional groups lead to the decreasing release rate of the drug from the pores of silica matrix. Muňoz et al. [18] studied the rate of drug release from the amino-modified MCM-41 silicas with different pore sizes. Their study showed that amino functionalization lead to decreasing the rate of drug release. It can be thought that this delivery rate could be tuned by modifying the interaction between the drug molecule and the silica matrix. Moreover, the organic functionalization of the mesoporous silica materials can influence their biocompatibility [19].

The encapsulation and controlled release of the therapeutic drug into/from the drug delivery material has become still more important for the therapeutic system. The main advantages of this system is especially safety and efficiency and take control over the negative influence of the using drug. Moreover, the drug can be loaded into the pores in a preferably amorphous state, which can increase the drug dissolution properties dramatically [20]. The most of anticancer drugs are known for their low solubility, low stability, short plasma half-life and cytotoxicity to normal cells [21]. One of the problematic drugs that have been widely used in anticancer chemotherapy is 5-fluorouracil.

**Figure 1:**
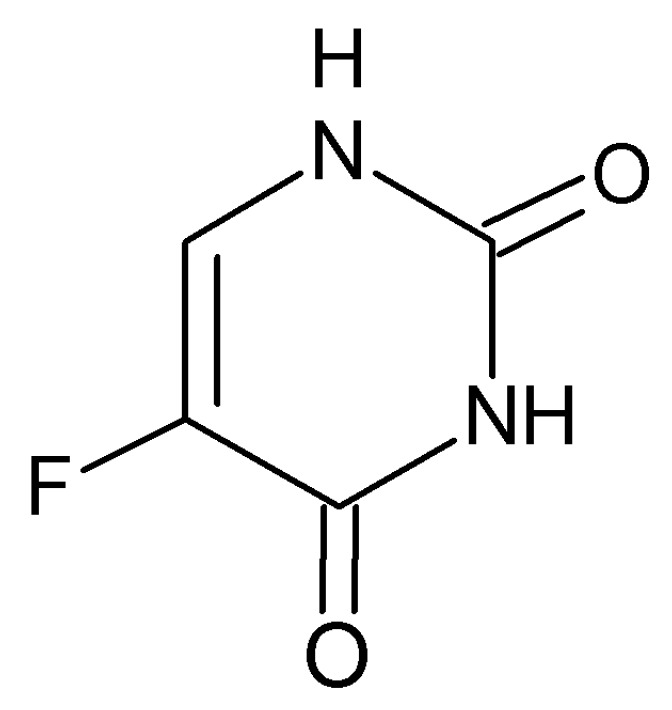
Chemical structure of 5-fluorouracil.

5-Fluorouracil (5-FU) is a water-soluble pyrimidine analogue (Figure 1), used in cancer treatment that has been particularly effective in the treatment of colorectal cancer [22,23], stomach, breast, and head and neck cancers [24,25]. Despite its water solubility, this antitumor agent suffers from several drawbacks: the oral use of 5-FU exhibits a short plasma half-life (30 min) as a result of its rapid enzymatic metabolism [26], which demands continuous high doses that simultaneously lead to high toxicity, incomplete and non-uniform oral absorption, the development of drug resistance by tumor cells, and nonselective action against normal cells [27,28].

As can be seen from the abovementioned facts, there are several limitations to the use of 5-FU, thus, encapsulation in an appropriate delivery system might contribute to reduce these side effects and improve its oral bioavailability, preventing its premature degradation and decreasing its side effects, increasing concentrations at the target cell site, with lower cytotoxicity for normal cells. Moreover, encapsulation may allow drugs to be released in a controlled way to the cancer area, preventing degradation of the anticancer drug [29]. Until now, controlled loading and releasing of 5-fluorouracil has been studied from several matrices: organic-modified montmorillonite [30], hybrid TiO_2_/ZnO nanotubes [31], layered inorganic nanocomposites [28], biodegradable polymers [32], hydrogels [33], mesoporous organosilica [34], magnetic nanocarriers [27], porous silica calcium phosphate nanocomposites [35] and zeolites [36,37]. To the best of our knowledge, there are only three reports on using silicate nanoparticles, namely thiol-functionalized MCM-41 [38], MCM-48 nanoparticles [39] and magnetic iron oxide/mesoporous silica nanocomposites (*m*-MCM-41) with and without aminopropyl functionalization [40], as carriers for 5-FU. In this work, we report encapsulation and subsequent release of 5-FU from silica SBA-15.

For the characterization of the delivery system such as mesoporous silica, suitable and validated quantitation method is required to assess pharmaceutical parameters such as drug content. Various methods for the determination of 5-FU have been reported, but mostly in biological fluids [41,42,43,44]. To the best of our knowledge, the study of controlled loading and releasing of 5-FU from mesoporous silica SBA-15 using reversed-phase ultra-high performance liquid chromatography RP-UHPLC has been not investigated, up to now.

Therefore, the objective of this work was to develop a fast, simple and optimized HPLC method to determine the encapsulation efficiency of 5-FU incorporated in unmodified and modified mesoporous silica SBA-15 and its quantitative analysis. In this study, SBA-15 material was functionalized with 3-aminopropyl and cyclohexyl groups by grafting method. The analysis of 5-FU released from unmodified and modified silica SBA-15 in defined time interval during 48 h was performed in two different media: simulated gastric fluid (pH = 2) and simulated body fluid (pH = 7). This study can be useful in the biomedical applications. The encapsulation and subsequent release of the 5-FU from mesoporous silica should decrease the negative influence of the drug using. The present work describes simple, rapid and precise chromatographic method based on RP-UHPLC mechanism for the desorption study of 5-fluorouracil from mesoporous silica SBA-15.

## 2. Results and Discussion

### 2.1. Characterization of SBA-15

**Figure 2:**
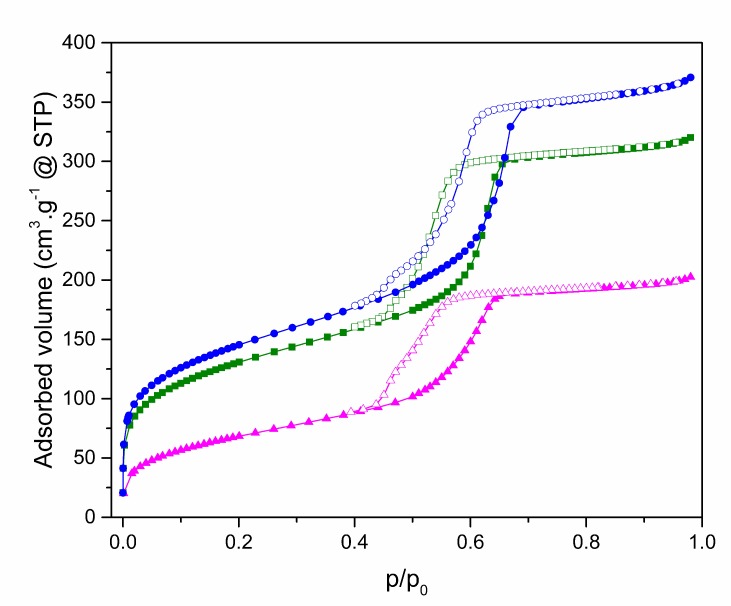
Isotherms of mesoporous silica SBA-15 before (blue) and after modification with 3-aminopropyl (cyan) and cyclohexyl (green) groups.

To evaluate the textural properties of prepared nanomaterials the method of nitrogen adsorption/desorption at 77 K was used. All measured isotherms are depicted at Figure 2.

**Figure 3:**
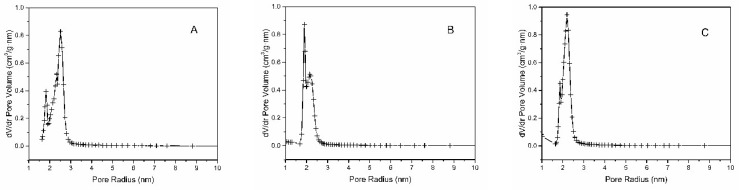
BJH pore size distribution for nanomaterials SBA-15 (**A**), nSBA-15 (**B**) and cSBA-15 (**C**) shows decrease in radius after modification step.

**Figure 4:**
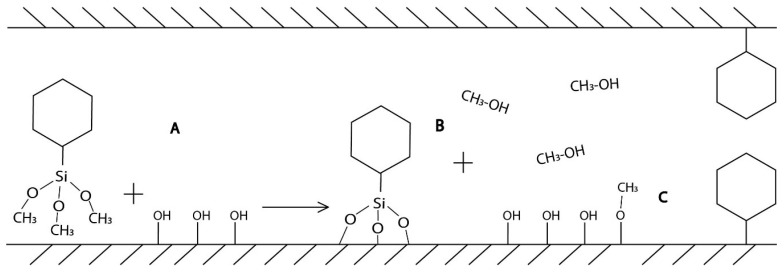
Schematic representation of possible reason for lower number of cyclohexyl groups in comparison to 3-aminopropyl groups. In the process of modification (**A**) the methanol was released as a by-product (**B**) and slowly diffuse into the reaction media. Alcohols can bind to the MPS surface and modify it (**C**), which results in the lower number of free hydroxyl groups.

Isotherms are characterized by the presence of hysteresis loops. According to their shape, they are classified as IVb isotherms type. This type of isotherms is characteristic of mesoporous materials with 2D structure as SBA-15 [45]. The pure SBA-15 is shown in blue at Figure 2. In the range of relative pressure 0–0.05, there is a rapid increase in the adsorbed volume, which is associated with the filling of the micropores. The gradual increase of relative pressure to 0.5 is accompanied by the steady increase of adsorbed volume caused by the multilayer formation. Capillary condensation, which is manifested by a sharp increase in the isotherm, occurs in the 0.6–0.7 relative pressure range. Above 0.7, no more N_2_ was adsorbed because the pores are already filled until 0.95 where is the slight rise of isotherms caused by the condensation of N_2_ on the external surface between nanoparticles. Desorption isotherms are shifted to lower relative pressures, which is characteristic nature of SBA-15 type of materials. The amount decreasing of adsorbed volume is proportional to the amount of bound organic groups on the surface and their bulkiness. All computed values are summarized in a Table 1. From the BJH graphs at Figure 3 is evident, that the nanomaterials are characteristic with narrow pore size distribution. Moreover, the nSBA-15 and cSBA-15 increase the number of pores with smaller radius. It is evident from the comparison of all computed values, that the least number of immobilized groups was attached to the surface of cSBA-15. This behaviour could be explained by the bulkiness and hydrophobic nature of cyclohexyl ligand in comparison to 3-aminopropyl group. Another possible explanation (Figure 4) is related to the type of precursor used in modification step. Cyclohexyltrimethoxysilane was used as a modifying agent (Figure 4A), which by bonding to the surface, releases methanol as a by-product (Figure 4B).

The binding of chemical groups occurs primarily on the external surface of the MPS, which is associated with a better diffusion of reaction products to the reaction medium. However, if the cyclohexyl group is bonded inside the pores, the methanol is repelled by the cyclohexyl groups bounded to the external surface. Moreover, according to Björklund and Kocherbitov [46], alcohols can bind to the surface of silica and modify it (Figure 4C), which results in less free hydroxyl groups for modification. This synergic effect can reduce the amount of immobilized cyclohexyl groups on the surface of MPS.

**Figure 5:**
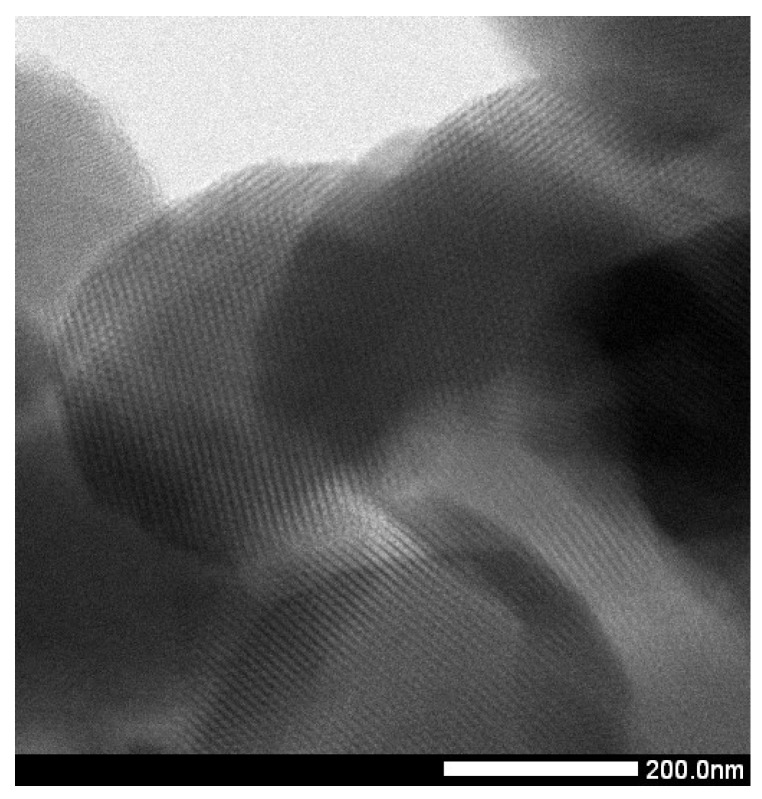
HRTEM micrograph of SBA-15.

A high-resolution transmission electron microscopy (HRTEM) micrograph was taken to prove the morphology of the prepared SBA-15 material. As can be seen at Figure 5, the mesoporous material SBA-15 has regular porous structure with a hexagonal array of uniform channels with average pore diameter about 5 nm. Average pore size suggested by HRTEM micrograph well agree with the result calculated by nitrogen adsorption/desorption measurement.

**Figure 6:**
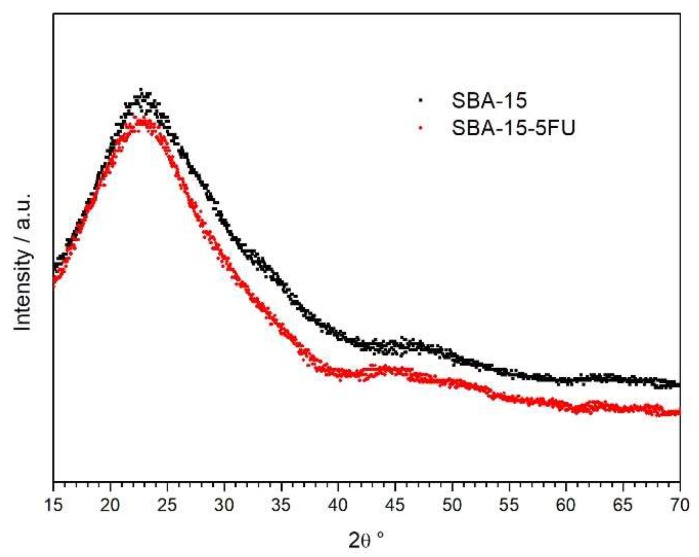
XRD pattern of samples SBA-15 (black) and SBA-15 with 5-fluorouracil loaded (red).

Mesoporous materials are stable, and their regular porous structure does not change upon drug adsorption. Regular porous structure and stability of SBA-15 silica and other mesoporous matrices was proven by Small Angle X-ray Scattering (SAXS) in our previous works [10,11,12,47,48,49,50]. Wide Angle X-ray Scattering (WAXS) or XRD measurements do not give useful information, as mesoporous silica matrices have amorphous walls, so no diffraction from the matrix is observed and at high angles (15 to 70°) the XRD patterns of SBA-15 and the 5-flourouracil loaded sample only showed a broad peak at 2θ = 23–26°, which is characteristic of amorphous silica, and the characteristic XRD peaks of crystalline 5-fluorouracil were not observed. This finding indicates a good dispersion of 5-FU inside the support mesopores (Figure 6).

This behaviour is one of the advantages of mesoporous materials as drug delivery systems, which enhances the solubility of hydrophobic drugs. For this reason, these materials are very suitable for delivery of drugs with low water solubility, like 5-fluorouracil [51]. The amount of loaded 5-FU in each sample was obtained by analysing the change in concentration of the solutions before and after the adsorption step. 5-FU was loaded into the pores of the samples by the penetration method. Prior to experiment, a 10 mg/mL 5-FU solution was prepared, and the exact concentration was measured. To 10 mL of 5-FU solution was added 100 mg of nanomaterial. Consequently, the largest amount that can be loaded into the samples is 1 gram of 5-fluorouracil per gram of nanomaterial (g_5-FU_/g). The loading capacity of pure SBA-15 is 142.3 mg_5-FU_/g. Nanomaterial nSBA-15 has a loading capacity equals to 184.25 mg_5-FU_/g. The observed gain can be explained by the improved interaction of 5-FU molecules with the modified surface via hydrogen bonds. The last sample cSBA-15 has loading capacity of 146 mg_5-FU_/g. The loading capacity of 5-FU was calculated as following Equation (1):

(1) $$5\text{-FU}\;\mathrm{loading}\;\mathrm{capacity}=\frac{\mathrm{weight}\;\mathrm{of}\;\mathrm{loaded}\;5\text{-FU}\;\mathrm{in}\;\mathrm{the}\;\mathrm{pores}\;}{\mathrm{weight}\;\mathrm{of}\;\mathrm{whole}\;\mathrm{delivery}\;\mathrm{system}}$$

The amount of drug loaded into the mesoporous samples was determined after subtracting the amount of unloaded drug from the initial amount of drug in loading medium. The exact concentration of 5-FU in media during sample preparation was measured using UV-VIS spectrophotometry.

The encapsulation efficiency for samples 5-FU-SBA-15, 5-FU-nSBA-15 and 5-FU-cSBA-15 was 14.23%, 18.425% and 14.6%, respectively. The encapsulation efficiency was calculated by the following Equation (2):

(2) $$\mathrm{encapsulation}\;\mathrm{efficiency}=\frac{\mathrm{weight}\;\mathrm{of}\;\mathrm{loaded}\;5\text{-FU}\;\mathrm{in}\;\mathrm{the}\;\mathrm{pores}}{\mathrm{initial}\;\mathrm{weight}\;\mathrm{of}\;5\text{-FU}\;\mathrm{in}\;\mathrm{loading}\;\mathrm{media}}\times 100\%$$

**Figure 7:**
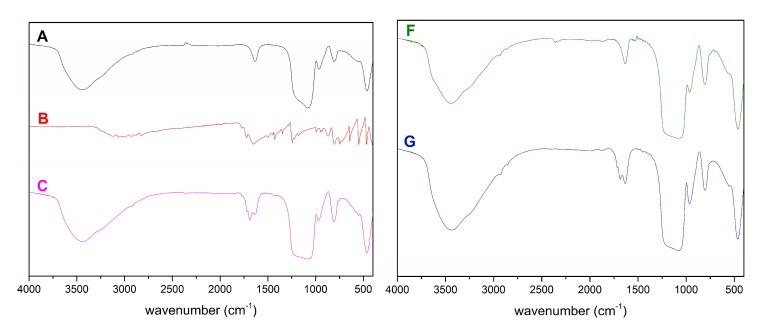

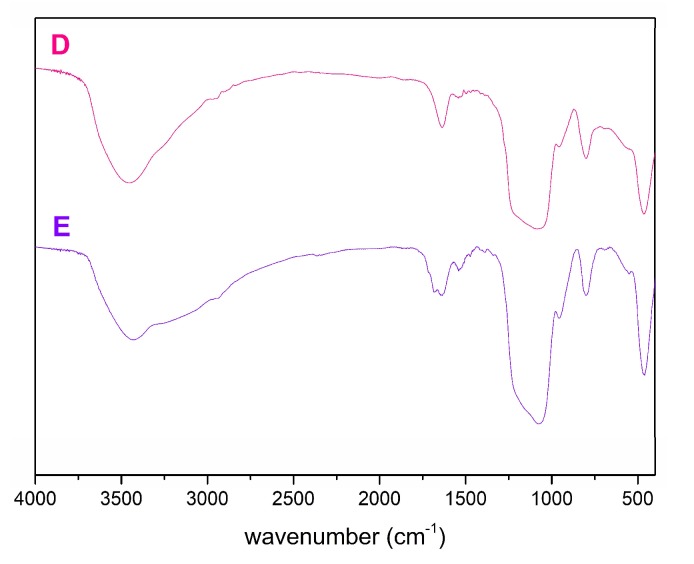
Infrared spectra of nanomaterials SBA-15 (**A**), 5-FU (**B**), 5-FU-SBA-15 (**C**), nSBA-15 (**D**), 5-FU-nSBA-15 (**E**), cSBA-15 (**F**) and 5-FU-cSBA-15 (**G**).

An infrared spectroscopy method of was used to verify the success of SBA-15 surface modification and subsequent adsorption of 5-fluorouracil. The measured infrared spectra are depicted in Figure 7.

The presence of a broad band in the 3750–3100 cm^−1^ range, which is assigned to the stretching vibrations of O-H and Si-OH bonds, is characteristic of all mesoporous silica samples. Free silanol groups on the surface of mesoporous silica can be detected around 960 cm^−1^. The presence of asymmetric vibration of the Si-O-Si group overlapping with the Si-O-C and Si-C vibrations is observed in the 1100–1200 cm^−1^ range. Another vibration is located around 790 cm^−1^, which corresponds to the symmetrical Si-O-Si bond stretch. Because all modifying agents used in this work are organic, characteristic vibrations of organic groups such as the stretching vibration of the C-H group in the 2900 cm^−1^ region or the bending vibration of the CH_2_ group in the 1520 cm^−1^ region are visible in the infrared spectra. Because the ligands are only bound to the surface, they occupy only a small portion of the mesoporous silica and the observed vibrations are therefore very weak, but strong enough to prove the success of surface modification. After loading of 5-FU into the pores the infrared spectrum of mesoporous systems slightly changed. Specifically, the most noticeable difference is presence of broader vibration (C, E, G) in the 1500–1750 cm^−1^ range. Most 5-FU is loaded into the pores of nSBA-15, which is evidenced by a broader and more intensive band between 2400–3400 cm^−1^ compared to clear nSBA-15 material.

### 2.2. Release Study of 5-Fluorouracil from Mesoporous Silica SBA-15 by RP-UHPLC

UHPLC with a photodiode array detector (PAD) was chosen as a simple, fast, and effective separation method for the determination of released 5-FU from studied mesoporous silica SBA-15 samples in defined time intervals (5 min, 15 min, 30 min, 1 h, 2 h, 4 h, 6 h, 24 h and 48 h). To optimize the operating conditions for isocratic RP-UHPLC detection of the released amount of 5-FU a number of parameters such as the column type, mobile phase composition, column temperature and the flow rate were varied. The effect of mobile phase composition on retention time of 5-FU was examined. For solvent A, the common reversed-phase solvents such as methanol and acetonitrile were compared. Methanol gave the best separation of the peaks. Phosphate buffer with pH = 6.9 was used for solvent B. The variations in mobile phase composition consisted of changes in the methanol to phosphate buffer (pH = 6.9) volume ratio. Good separation of 5-FU and short run time were obtained by using a mobile phase consisted of methanol: phosphate buffer (pH = 6.9) in volume ratio of 3:97 (*v*/*v*). Optimized chromatographic conditions for determination of released amount of 5-FU from mesoporous silica are summarized in Table 2. These chromatographic conditions were the same for both media: simulated gastric fluid (pH = 2), as well as simulated body fluid (pH = 7).

**Figure 8:**
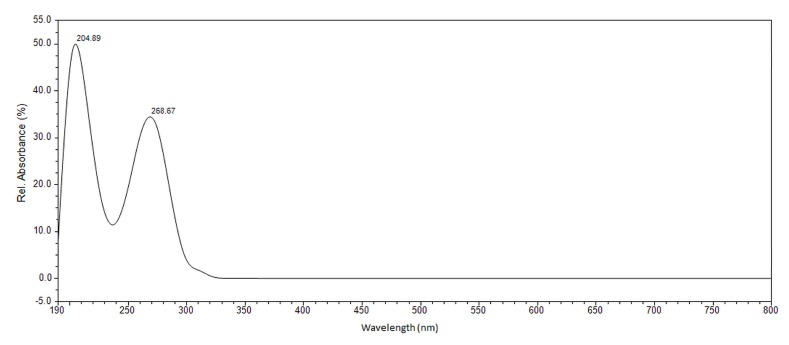
Absorption spectrum of 5-FU.

The absorption spectrum of the drug was recorded between 190 and 800 nm. Peak identification was done by comparing the retention times and absorption spectra of the samples with 5-FU standard. The optimal absorption wavelength for 5-FU was determined by measuring the standard solutions. 5-FU exhibits two absorption maxima at 204 and 268 nm (Figure 8). UV radiation below 200 nm (vacuum area) is not used routinely because of the technical complexity of such measurements. Radiation in this region is strongly absorbed by atmospheric oxygen, thus the measurement of the absorption spectra near the 200 nm wavelength is very problematic. Therefore, a second absorption band with a wavelength 268 nm was selected as optimal for determination of the 5-FU released amount.

**Figure 9:**
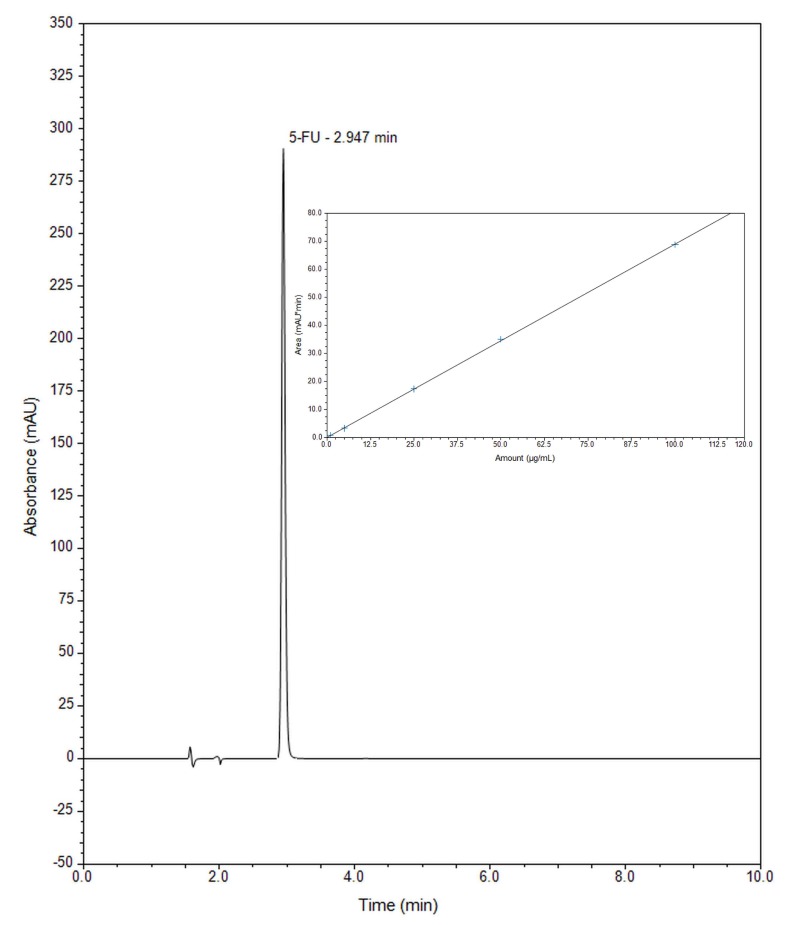
Chromatogram of 5-FU standard solution (25 µg/mL) and calibration curve for 5-FU.

Under the experimental conditions, the chromatogram of a standard solution of 5-FU, with detection at the absorption maximum of 5-FU is shown in Figure 9, where the concentration of 5-FU is 25 µg/mL. 5-FU standard solution shows one peak at a retention time of 2.9 min.

The released amount of 5-FU was determined by plotting the calibration curve (Figure 9). Standard solutions of 5-FU were prepared and analysed with three replicates and the results were processed with the Chromeleon Version 7.2 software. The calibration curve was constructed by plotting the peak area against each 5-FU concentration for the five standard solutions in water. Under the above-described experimental chromatographic conditions, linear correlation between the peak area and applied concentration was found in the concentration range 1–100 µg/mL, as confirmed by the correlation coefficient of 0.99996. The value of correlation coefficient near 1 indicates very good linearity in the proposed range. The peak area (y) is proportional to the concentration of released 5-FU (x) following the regression equation y = 0.6892 x + 0.1062. The sensitivity of HPLC method was determined by LOD and LOQ. LOD is the lowest concentration of the analyte the method can detect and LOQ is the lowest concentration that can be quantified accurately by the method. The experimentally derived LOD and LOQ for 5-FU were determined to be 0.1134 and 0.3742 µg/mL, respectively.

**Figure 10:**
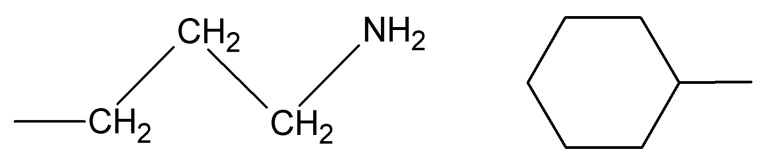
Chemical structure of the 3-aminopropyl (left) and cyclohexyl (right) groups.

5-FU was encapsulated into the pores of silica SBA-15 and consequently released out of the structure of this matrix during 48 h. The surface of mesoporous silica SBA-15 was modified with various organic groups, such as 3-aminopropyl group (nSBA-15) and cyclohexyl group (cSBA-15) (Figure 10).

**Figure 11:**
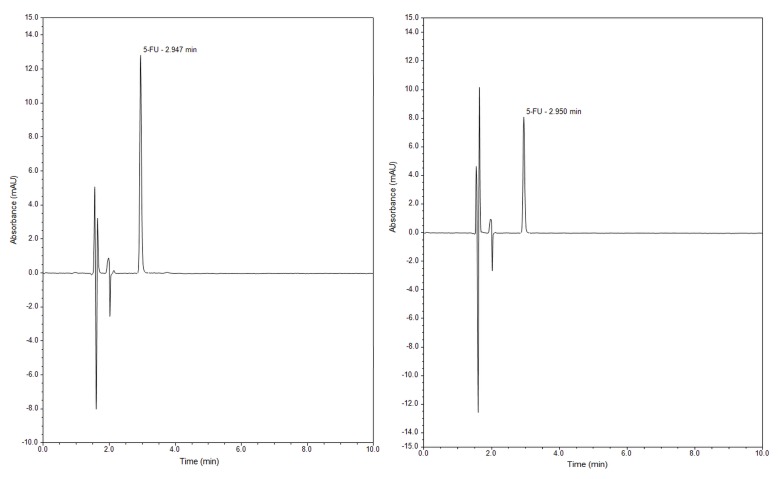
Representative chromatogram of 5-FU released during 4 h from SBA-15 into medium with pH = 2 (left) and medium with pH = 7 (right).

The amounts of the drug released from samples were monitored in selected time intervals ranging from 5 min to 48 h by RP-UHPLC in two different media, which are as follows: simulated gastric fluid represented by buffer solution with pH = 2 and simulated body fluid represented by physiological solution with pH = 7. Under the optimized conditions of the present assay, the drug was eluted with optimum resolution at about 2.9 min in acidic and neutral medium. Figure 11 shows typical chromatograms of a working sample solution obtained under the optimized HPLC conditions described in the preceding for both media.

**Figure 12:**
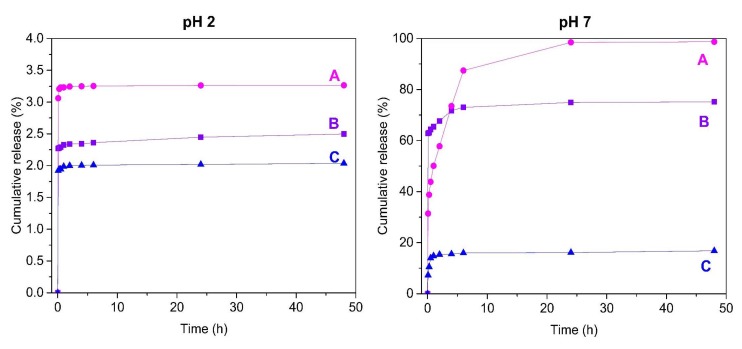
Release profiles of 5-fluorouracil from 5-FU-SBA-15 (**A**), 5-FU-nSBA-15 (**B**) and 5-FU-cSBA-15 (**C**) nanomaterials into the media with different pH.

According to the chromatographic results, the curves of the time dependences of 5-FU were constructed. As can be seen from Table 3 and Figure 12, the release profile of 5-FU from prepared mesoporous nanomaterials is strongly dependent on the pH of the release media. Release of 5-FU to an acidic pH environment practically did not occur, as only up to 3.26% was released for unmodified SBA-15. A different situation occurs with the release of 5-fluorouracil in physiological saline. A higher pH significantly improved the solubility of 5-FU. Unmodified SBA-15 material interacts with 5-FU molecules only by the silanol groups on the surface, so nearly all 5-FU was released. Surface modification by 3-aminopropyl groups improve the wettability of the nanomaterial, so the release of 5-FU was faster in comparison to unmodified SBA-15. Moreover, interaction of 5-FU with 3-aminopropyl groups leads to lower released amounts. On the other hand, modification with cyclohexyl groups increases the hydrophobicity. Nanomaterial cSBA-15 cannot mix well with the release medium so the release occurs only from the external surface. All samples are characterized by their fast release profiles. Similar release profiles of 5-FU from mesoporous silica were observed in earlier studies. Egodawatte et al. studied the influence of the solvent in the preparation of 5-FU containing magnetic mesoporous nanoparticles and all release profiles were characterized by a fast initial release of 5-FU [40].

The pH-dependent release behaviour of the prepared nanocarriers has many advances compared to free 5-FU. 5-FU is commonly given intravenously because of the complications associated with its oral administration. 5-FU is anticancer drug for treatment of various tumours such as colon, stomach and lung [52]. Using mesoporous silica as drug carrier can improve the site specificity of 5-FU, for example, in colon cancer treatment. In gastrointestinal fluid with low pH, 5-FU stay in pores until the drug delivery system reaches neutral colon pH conditions.

## 3. Materials and Methods

### 3.1. Instruments

Chromatographic separation was carried out using a Dionex UltiMate 3000 RS HPLC system equipped with a Chromeleon Chromatography Data System, Version 7.2 (Thermo Fisher Scientific, Braunschweig, Germany) comprising a quaternary pump, degasser, automated injector, column oven, and photodiode array detector (PAD). Analyses was performed on a chromatographic column (150 × 4.6 mm) filled with the sorbent ODS Hypersil C18 with particle size of 5 µm (Thermo Fisher Scientific). A pH-meter inoLab pH7110 (WTW, Weilheim, Germany) equipped with a combined electrode SenTixR 41 (WTW, Weilheim, Germany) was employed for pH measurements. Syringe filters PVDF 0.45 µm (Fisher Scientific, Pardubice, Czech Republic) were used for solvents and sample filtration throughout the experiment. Infrared spectra of prepared samples were measured using Thermo Fisher Scientific Nicolet 6700 FT-IR spectrophotometer (Thermo Fisher, Madison, WI, USA). All FT-IR measurement was done using KBr pellet method. The concentration of 5-FU in media during sample preparation was measured on Spekord 350 UV-VIS spectrophotometer made by Analytic Jena (Jena, Germany). The texture characterization of mesoporous silica by adsorption/desorption of N_2_ at 77 K was done using a Micromeritics ASAP 2020 plus instrument (Norcross, GA, USA). To calculate the specific surface area of prepared nanomaterials the Brunauer-Emmett-Teller (BET) equation was used. Volume of pores was compute using t-plot method and the pore size are calculated using Barrett-Halenda-Joyner method (BHJ). HRTEM images were taken with a JEOL TEM 2100F UHR microscope (Tokio, Japan) operated at 200 kV. A powder sample was sonicated in ethanol for 10 min. Copper grid covered with carbon was used as a support for sample.

### 3.2. Chemicals and Reagents

All reagents used, such as orthophosphoric acid 85% EMSURE^®^, potassium dihydrogenphosphate and disodium hydrogenphosphate dodecahydrate (Merck, Darmstadt, Germany), tetraethylorthosilicate (TEOS), cyclohexyltrimethoxysilane (CHTMS) (99%, pK_A_ = 7.55), (3-aminopropyl)triethoxysilane (APTES, 99%, pK_B_ = 3.63), poly(ethylene oxide)-poly(propylene oxide)-poly(ethylene oxide) triblock copolymer (Pluronic P-123) and 5-fluorouracil (5-FU, ≥ 99% by HPLC) (Sigma-Aldrich, St. Louis, MO, USA) were at least analytical grade and no further purification was done. Buffer solution with pH = 2 (hydrochloric acid/potassium chloride), according to Veibel was purchased from Honeywell Fluka (Steinheim, Switzerland) and physiological saline with pH = 7 was purchased from Centralchem (Banská Bystrica, Slovak Republic). Toluene (>99%) was purchased from Sigma-Aldrich and dried over zeolite sieves. Methanol LiChrosolv^®^ was of gradient grade for liquid chromatography (Merck). Water for chromatography LiChrosolv^®^ (Merck) was used for the preparation of a mobile phase and all solutions.

### 3.3. Preparation of Samples and Standard Solutions

SBA-15 was used as a model mesoporous silica with narrow distribution of pore size and uniform structure. Preparation of SBA-15 material was done by the modified sol-gel process invented by Zhao et al. [5]. Specifically, SBA-15 was prepared by dissolving Pluronic P-123 (8 g) as a structure-directing agent in deionized water (60 g) and adding 2 M HCl (240 g) with continuous stirring (400 rpm) and constant temperature (35 °C) conditions. Then TEOS (17 g) was added dropwise to the mixture after complete dissolution of the Pluronic P-123. Than the mixture was left to stir at 400 rpm under constant temperature (35 °C) for 20 h. Subsequently, the mixture was moved to the furnace heated to 80 °C and left to mature for another 24 h. After that step, the solid product was filtered off, washed out with deionized water and left to dry at laboratory conditions. The final nanomaterial was obtained by calcination using slow temperature rise rate (1 °C/min) and two isothermal steps at 100 °C (3 h) and 550 °C (7 h) followed by slow cooling to laboratory temperature.

Modified SBA-15 was prepared by a grafting method. Prior to modification the SBA-15 was dried at furnace set to 150 °C for 3 h. Subsequently, the SBA-15 (0.5 g) was dispersed in dry toluene (50 mL) in a 100 mL flask and briefly ultrasonicated to break the silica clots into a uniform mixture. Next, APTES or CHTMS (3 mL) was added dropwise to the mixture and left to react under reflux for 24 h. The solid modified product was filtered off and washed with toluene. Soxhlet extraction with toluene as extraction medium was used to remove unreacted modification agent from the mesoporous silica. Finally the material was dried and denoted as nSBA-15 (modified with 3-aminopropyl groups) and cSBA-15 (modified with cyclohexyl groups), respectively.

The impregnation method was used to prepare mesoporous silicas containing 5-FU. 100 mg of each prepared material was immersed in 10 mL of a 10 mg/mL 5-fluorouracil aqueous solution and allowed to mix in closed container under laboratory conditions for 24 h. After 24 h each sample was centrifuged, and 1 mL sample was taken for concentration analysis. Subsequently, the all solid samples were vacuum filtered and dried under vacuum. Prepared samples were denoted as 5-FU-SBA-15, 5-FU-nSBA-15 and 5-FU-cSBA-15.

A stock standard solution of 1.0 mg/mL of 5-FU was prepared in water and subsequent dilutions were carried out to obtain five standard solutions (1.0, 5.0, 25.0, 50.0, 100.0 μg/mL). The standards and samples had previously been filtered through a syringe filter with a pore diameter of 0.45 µm prior to injection and chromatographed.

### 3.4. Release Study of 5-Fluorouracil from Mesoporous Silica by RP-UHPLC and Chromatographic Conditions

The release of drug from mesoporous silica nanocarriers was studied in two different media: a buffer solution with pH = 2 and a physiological saline solution with pH = 7. Specifically, 10 mg of each sample was immersed in 50 mL of release medium at constant stirring (300 rpm) and temperature (37 °C) conditions. The amount of the 5-FU released from the mesoporous silica SBA-15 was analysed using RP-UHPLC in defined time intervals. To determine the release profile, sampling for chromatographic analysis was performed at predetermined intervals after 5 min, 15 min, 30 min, 1 h, 2 h, 4 h, 6 h, 24 h and 48 h. ODS Hypersil C18 column (150 × 4.6 mm, 5 µm) was used as stationary phase. The HPLC operating mode was isocratic, the column temperature 25 °C. The mobile phase contains a mixture of methanol: phosphate buffer adjust to pH = 6.9 (3:97, *v*/*v*), which was pumped at a flow rate of 1.0 mL/min. The PAD detector was set to collect signals within the spectral range of 190–800 nm. The effluent was monitored at 268 nm and injection volume was set at 20 µL. The samples were filtered through a syringe filter with a pore diameter of 0.45 µm and chromatographed. Optimized chromatographic conditions for determination of released amount of 5-FU from mesoporous silica are summarized in Table 1. Data acquisition, analysis, and reporting were performed using the Chromeleon Chromatography software, Version 7.2 (Thermo Fisher Scientific, Braunschweig, Germany).

## 4. Conclusions

An RP-UHPLC method for the determination of released 5-FU from studied mesoporous silica SBA-15 samples in defined time intervals has been developed and is presented here. The release amount of the drug was studied as a function of time in two different media, which are as follows, simulated gastric fluid represented by buffer solution with pH = 2 and simulated body fluid represented by physiological solution with pH = 7. The method uses isocratic elution and is shown to be selective, providing good accuracy and sensitivity. It was found out that surface functionalisation of silica SBA-15 influences the release properties of the drug. The functionalisation of the surface with more bulky ligands such as cyclohexyl led to lower drug release. According to the chromatographic results, larger amount of the drug was released in neutral pH in comparison with the acidic medium.

## Figures and Tables

**Table 1:** Textural characteristics of SBA-15 mesoporous drug nanocarriers (S_BET_—specific surface area, D_P_—pore diameter, V_P_—volume of pores).

Material	S_BET_ (m^2^/g)	D_P_ (nm)	V_P_ (cm^3^/g)
SBA-15 ^1^	528	5	0.496
nSBA-15 ^2^	250	4.7	0.289
cSBA-15 ^3^	477	4.9	0.435

^1^ SBA-15: unmodified silica; ^2^ nSBA-15: modified with 3-aminopropyl groups; ^3^ cSBA-15: modified with cyclohexyl groups.

**Table 2:** Optimized chromatographic conditions for determination of released amount of 5-FU from mesoporous silica SBA-15.

Parameters	Conditions
Mobile phase	methanol: phosphate buffer, pH 6.9 (3:97, *v*/*v*)
Stationary phase	ODS Hypersil C18 column, (150 × 4.6 mm, 5 µm)
Column Temperature	25 °C
Detection Wavelength	268 nm
Flow rate	1.0 mL/min
Injection volume	20 µL
Retention time	2.9 min

**Table 3:** Cumulative release of 5-FU from the samples in media with different pH.

	Cumulative Release of 5-FU (%)					
Time	pH 2			pH 7		
	SBA-15	nSBA-15	cSBA-15	SBA-15	nSBA-15	cSBA-15
5 min	3.06	2.26	1.92	31.40	62.82	7.27
15 min	3.20	2.27	1.94	38.77	63.09	10.53
30 min	3.23	2.29	1.94	43.83	64.34	13.94
1 h	3.23	2.32	1.98	50.08	65.43	14.82
2 h	3.24	2.33	1.99	57.76	67.68	15.35
4 h	3.24	2.34	2.00	73.51	71.74	15.57
6 h	3.25	2.36	2.00	87.42	72.99	15.94
24 h	3.26	2.44	2.01	98.48	74.93	16.13
48 h	3.26	2.49	2.03	98.69	75.16	16.81
